# Clinical Application of Metagenomic Next-Generation Sequencing for Suspected Infections in Patients With Primary Immunodeficiency Disease

**DOI:** 10.3389/fimmu.2021.696403

**Published:** 2021-08-13

**Authors:** Wenjing Tang, Yu Zhang, Chong Luo, Lina Zhou, Zhiyong Zhang, Xuemei Tang, Xiaodong Zhao, Yunfei An

**Affiliations:** ^1^Department of Rheumatology and Immunology, Children’s Hospital of Chongqing Medical University, Chongqing, China; ^2^Ministry of Education Key Laboratory of Child Development and Disorders, Children’s Hospital of Chongqing Medical University, Chongqing, China; ^3^National Clinical Research Center for Child Health and Disorders, Children’s Hospital of Chongqing Medical University, Chongqing, China; ^4^China International Science and Technology Cooperation Base of Child Development and Critical Disorders, Children’s Hospital of Chongqing Medical University, Chongqing, China; ^5^Chongqing Key Laboratory of Child Infection and Immunity, Children’s Hospital of Chongqing Medical University, Chongqing, China

**Keywords:** metagenomic next-generation sequencing, infection, diagnosis, primary immunodeficiency disease, conventional microbiological tests

## Abstract

**Background:**

Infections are the major cause of morbidity and mortality in patients with primary immunodeficiency disease (PID). Timely and accurate microbiological diagnosis is particularly important in these patients. Metagenomic next-generation sequencing (mNGS) has been used for pathogen detection recently. However, few reports describe the use of mNGS for pathogen identification in patients with PID.

**Objective:**

To evaluate the utility of mNGS for detecting pathogens in patients with PID, and to compare it with conventional microbiological tests (CMT).

**Methods:**

This single center retrospective study investigated the diagnostic performance of mNGS for pathogens detection in PID patients and compared it with CMT. Sixteen PID patients with suspected infection were enrolled, and medical records were analyzed to extract detailed clinical characteristics such as gene variation, immune status, microbial distribution, time-consuming of mNGS and CMT, treatment, and outcomes.

**Results:**

mNGS identified pathogenic microbe in 93.75% samples, compared to 31.25% for culture and 68.75% for conventional methods, and detected an extra 18 pathogenic microorganisms including rare opportunistic pathogens and *Mycobacterium tuberculosis*. Pathogen identification by mNGS required 48 hours, compared with bacterial culture for 3-7 days and even longer for fungus and *Mycobacterium tuberculosis* culture.

**Conclusions:**

mNGS has marked advantages over conventional methods for pathogenic diagnosis, particularly opportunistic pathogens and mixed infections, in patients with PID. This method might enable clinicians to make more timely and targeted therapeutic decisions, thereby improving the prognosis of these patients.

## Introduction

Primary immunodeficiency disease (PID) or inborn errors of immunity are caused by monogenic mutations, resulting in loss- or gain-of-function of the encoded protein. They manifest as increased susceptibility to infectious diseases, as well as a growing diversity of autoimmune, autoinflammatory, allergic, lymphoproliferative, and/or malignant phenotypes. They comprise 404 distinct disorders, with 430 different gene defects (1, 2). Infection is a major cause of repeated hospitalization and eventual death in children with PID. A report from France shows that 85% of non-transplant PID patients were admitted to hospital related to acute infections (3). Timely and effective anti-infection therapy is of great importance for reducing infection mortality and improving transplant success rates in PID patients.

Metagenomic next-generation sequencing (mNGS) has been used in many fields in recent years; this technology allows simultaneous and independent sequencing of thousands to billions of DNA fragments, thereby facilitating an unbiased approach to broad identification of both known and unexpected pathogens (or even the discovery of new organisms). Compared with culture-based methods, mNGS offers advantages such as short turn-around times and unbiased quantitative or semi-quantitative analysis. In addition, multiple agents across the full microbial spectrum can be detected simultaneously by mNGS, along with identification of non-culturable microbes (4). The cost of mNGS has fallen by several orders of magnitude since its advent in 2004; it has emerged as an enabling technological platform for detection of microorganisms in clinical samples. Recent studies used mNGS to identify pathogens in the respiratory and central nervous systems, and pathogens that cause focal and bloodstream infections, and pathogens in immunosuppressed patients (5–14). However, only a few studies report the use of mNGS to detect infectious agents in patients with PID (15–17). Due to the unique characteristics and overall severity of infections in these patients, rapid and accurate diagnostic methods are needed urgently. This study aimed to determine whether mNGS technology can meet this need by assessing its ability to detect pathogens in patients with PID, and by comparing its efficacy with that of conventional microbiological tests (CMT).

## Materials and Methods

### Study Design and Participants

This study comprised a retrospective analysis of data from 16 PID patients with suspected infections admitted to Children’s Hospital of Chongqing Medical University in Chongqing, China from October 2018 to December 2020. Samples from these patients were subjected to both CMT and mNGS. Extensive phenotype information, including clinical and laboratory data, were available for all patients to facilitate interpretation of results. Infections were suspected in patients presenting with symptoms such as fever, cough, weakness, headache, hemiplegia, abdominal pain, and ostealgia. The study was approved by the Medical Ethics Committee of the Children’s Hospital of Chongqing Medical University and was conducted in accordance with The Code of Ethics of the World Medical Association (Declaration of Helsinki). All patients provided written informed consent prior to sample collection.

### Specimen Collection and Processing

During enrollment, samples from all subjects underwent CMT ordered by their treating clinicians. Different clinical specimens were collected for testing based on the type of suspected infection (i.e., cerebrospinal fluid (CSF), bronchoalveolar lavage fluid (BALF), peripheral blood, sputum, liver biopsy tissue, pus, and bone biopsy tissue). The CMT included blood/sputum/BALF/CSF cultures, serological tests, molecular diagnostic tests, and antigen detection. CMT were conducted in accordance with a clinical assessment of necessity. At the same time, additional samples were collected and transported overnight to Kindstar Global Laboratories (Wuhan, China) for mNGS.

### Sample Processing and Library Construction

Before nucleic acid extraction, samples were processed as follows: tissue samples were ground into a homogenate, and sputum was liquefied. DNA was extracted with TIANamp Micro DNA Kit (TIANGEN BIOTECH, Beijing, China) from collected samples according to the manufacturer’s instructions. Next, libraries were constructed for NGS, as described previously (13). When patients underwent mNGS, due to sample volume, test price and other factors, no relevant information about RNA viruses was collected from all patients included in this study.

### Next Generation Sequencing

Samples were transported to Kindstar Global Wuhan for PMseqTM library construction and Illumina MiniSeq platform or Illumina NextSeq 550 platform for high-throughput metagenomic sequencing. The minimum limit of microbe detection of this technique is 100 copies/mL (viruses = 1000 copies/mL). The specificity and repeatability of microbial detection were both > 99% when the copy number was above the minimum limit of detection.

### Analysis of Sequencing Data

As described previously, adapter contamination, low quality and low-complexity reads were quality filtered using an in-house program (13). Next, Burrows-Wheeler Alignment (Version: 0.7.10) was used to map the filtered sequences to a human reference database that includes hg38 and the Yanhuang genome sequence. The Remaining data were classified into four Microbial Genome Databases: viruses, bacteria, fungi, and parasites. Classification reference databases were downloaded from NCBI (ftp://ncbi.nlm.nih.gov/genomes/). The depth and coverage of each species were calculated by Soap Coverage software from the SOAP website (http://soap.genomics.org.cn/). Next, the parameter values were normalized according to data size and detected species listed in the suspected background database were filtered, as previously reported (13, 18). Based on the relative abundance of microbes detected by NGS in healthy control samples, we set up the threshold for each microbe to allow further validation. Pathogens with the highest absolute abundance within their genus, and pathogens ranked in the top 10 fungi, viruses, and parasites, and ranked in the top 50 bacteria (in terms of relative abundance after the previous two screening steps), were selected. With respect to intracellular bacteria (i.e., *Mycobacterium tuberculosis* and *brucella*) and some fungi (i.e., *cryptococcus*), as long as the test data are compared with the above reference genomes, it is necessary to consider whether they are infectious pathogens in combination with clinical practice. If detected pathogens were common infectious pathogens, they were considered to be causative agents. In the case of uncommon pathogens, the mNGS results were interpreted in the context of the patient’s clinical features; otherwise, the detected reads were classified as “non-pathogenic” microbe sequences. Strictly map reads number (SMRN) and genomic coverage were analyzed. SMRN represents the number of sequences that are strictly aligned with the microorganism (genus/species), which can reflect the pathogenicity of detected pathogens to some extent. SMRN are affected by content of pathogen in the sample, the size of pathogen genome, the amount of nucleic acid extracted from the sample, and other factors. High SRMN in an detected pathogen does not completely mean it is pathogenic and vice versa. Genomic coverage refers to the percentage of the nucleic acid sequence length of the microorganism detected to the genome sequence length of the microorganism. Generally speaking, the higher the genomic coverage, the higher the credibility of the pathogen detected. But it also influenced by the type of pathogen.

### Statistical Analysis

Due to the small number of patients enrolled, differences among groups were analyzed using a two-tailed independent-samples t test. A P value <0.05 was considered significant. Data analysis was performed using GraphPad Prism software (GraphPad Software, San Diego, CA).

## Results

### Basic Clinical Information

Sixteen patients with PID (age range, 4 months to 16 years) were enrolled. Specific primary diseases were as follows: X-linked-hyper IgM syndrome (XHIM) caused by a *CD40LG* mutation (n=4, P1 to P4); Wiskott-Aldrich syndrome (WAS) caused by a *WASP* mutation (n=2, P5 and P6); CTLA4 deficiency(P7); PSTPIP1-associated myeloid-related proteinemia inflammatory syndrome (PAMI) caused by a *PSTPIP1* mutation (P8); X-linked lymphoproliferative disease (XLP) caused by a *SH2D1A* mutation (P9); NEMO deficiency caused by a *IKBKG* mutation (P10); chronic granulomatous disease (CGD) caused by a *CYBB* mutation (n=2, P11 and P12); Artemis deficiency caused by a *DCLRE1C* mutation (P13); Mendelian susceptibility to mycobacterial disease (MSMD) caused by a *STAT1* AD loss-of-function mutation (P14); X-linked agammaglobulinemia (XLA) caused by a *BTK* mutation (P15); and activated phosphoinositide 3-kinase-δ syndrome (APDS) caused by a *PIK3CD* AD GOF mutation (P16). The basic information, including age, gender, infection data, immunological characteristics, and gene variation are shown in **Supplementary Table 1**.

**Figure 1:**
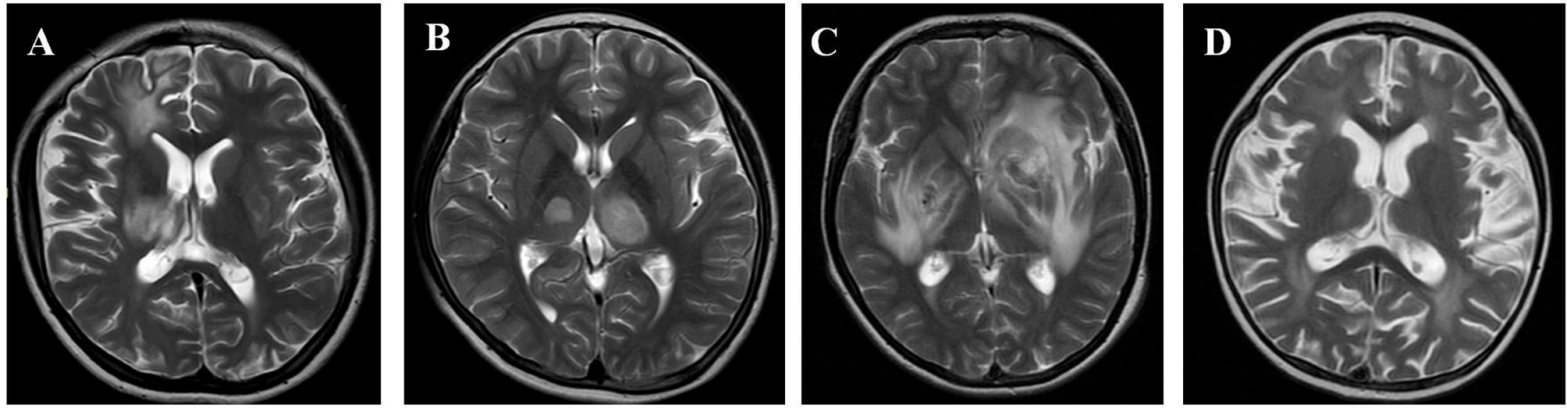
Cranial magnetic resonance imaging of patients. **(A)** Patient P1: Multiple abnormal bilateral signals in the brain. Both old and new lesions coexist. Demyelinating lesions may be present. **(B)** Patient P2: Sparse abnormal bilateral signals in the brain parenchyma, suggestive of inflammatory lesions. **(C)** Patient P3: Abnormal signals in the brain and cerebellum, particularly in the bilateral basal ganglia and the left frontal lobe, suggestive of infective lesions. **(D)** Patient P15: Abnormal, bilateral symmetrical signals in the white matter, brain atrophy, and similar signals in the thalamus.

**Figure 2:**
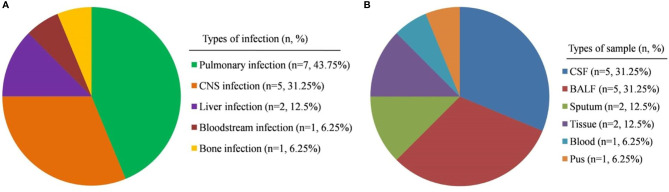
Composition of patients and samples types. **(A)** Type of suspected infection in the PID patients included in this study. **(B)** Types of sample collected in this study. CNS, central nervous system; CSF, cerebrospinal fluid; BALF, bronchoalveolar lavage fluid.

The clinical presentations of patients at the time of sample collection were as follows: patient P1 had claudication of the left lower extremity, weakness of the left hand, and intermittent fever; P2 presented mainly with intentional tremor of the right upper limb, unsteady gait, and occasional fever; P3 suffered dizziness, headache, vomiting, drowsiness, slurred speech, unsteady gait, and fever; P15 presented mainly with unstable gait, choking, and intermittent fever; and P16 presented mainly with fever and headache. The above five patients were suspected to have central nervous system (CNS) infections; therefore, cerebrospinal fluid (CSF) was collected and cranial images taken (**Figure 1**). Seven patients (P4, P7 to P11, and P13) all had cough and fever and so were suspected to have pneumonia; therefore, bronchoalveolar lavage fluid (BALF) or sputum was collected. P5 presented with fever and rash; we suspected sepsis and so obtained peripheral venous blood samples. P6 had abdominal pain and liver space-occupying lesions; liver biopsy tissues were collected and checked for specific pathogen infection or tumors. In patient P12, abdominal pain and fever were the main symptoms; abdominal ultrasound indicated that the main lesion was in the parenchymal components of the right lobe of the liver. The patient’s underlying disease was CGD. Therefore, liver abscess was considered and he received imipenem cilastatin and linezolid. After abdominal ultrasound showed fluid lesions, the liver abscess was punctured, drainage was performed, and the pus was collected. In patient P14, the main manifestations were abnormal gait, which progressed gradually to ostealgia in the head, chest, waist, and lower limbs, accompanied by intermittent fever. CT revealed multiple areas of bone destruction throughout the body, including the left humerus, left ulnar radius, right femur, right tibia, left fibula, frontal bone, parietal bone, occipital bone, and vertebral bodies C1–2, L1–3, and S1. Considering his primary disease, we suspected infection with pathogens such as *Mycobacterium*. A bone biopsy was performed and bone biopsy tissue was collected. The suspected infections and samples collected are shown in **Figure 2**.

### Diagnostic Performance of mNGS

The mNGS sequencing results for 15/16 (93.75%) patients were positive for microbial pathogens (**Table 1**). A comparison of the diagnostic results from mNGS with CMT is shown in **Table 2**. CMT and mNGS results were concordant for 11 of 15 patients (73.3%). However, agreement between the culture method and mNGS was only 33.3%. Positive agreement between mNGS and clinical diagnosis was significantly higher than that of culture and CMT (93.75% *vs.* 31.25% and 68.75%, respectively). In addition, mNGS detected 18 more pathogens than conventional methods in these patients.

**Table 1:** Results of CMT and mNGS of various samples for patients with PID.

Patients	Specimen for mNGS	Culture results	Other microbiogical testing results	mNGS results (pathogenic)	SMRN	Genomic coverage (%)
P1	CSF	Negative (CSF and blood)	Negative	*JC polyomavirus*	1526	97.563
				*BK Virus*	20	30.063
				*Acidovorax temperans*	3301	11.166
				*Comamonas testosteroni*	591	1.453
				*Malassezia globosa*	119	0.168
				*Acanthamoeba triangularis*	890	0.241
P2	CSF	Negative (CSF and blood)	Negative	*JC polyomavirus*	5	14.6
				*Acidovorax temperans*	30	0.119
				*Ralstonia mannitolilytica*	18	0.053
P3	CSF	Negative (CSF and blood)	Negative	*Toxoplasma gondii*	151	0.01
P4	sputum	Negative (sputum and blood)	CMV-DNA (+)	*Acinetobacter junii*	567	1.44
				*Stenotrophomonas maltophilia*	118	0.21
				*Pneumocystis jirovecii*	2	0.0018
				*Cytomegalovirus*	2	0.064
P5	blood	Negative (blood)	CMV-DNA (+)	*Cytomegalovirus*	18	1.139
P6	Liver tissue	Negative (blood)	EBV-DNA(+)	*Epstein-barr virus*	18	0.757
P7	BALF	*Klebsiella pneumoniae* (BALF)	Negative	*Klebsiella pneumoniae*	18957	3.0
P8	BALF	Negative (BALF and blood)	Mycoplasma pneumoniae (+)	*Epstein-barr virus*	45	2.781
				*Mycoplasman pneumonia*	154	2.479
P9	BALF	*Streptococcus pneumoniae*	Negative	*Streptococcus pneumonia*	67	0.434
		*Pseudomonas aeruginosa*		*Pseudomonas aeruginosa*	6	0.014
		(BALF)		*Haemophilus influenza*	3	0.024
				*Epstein-barr virus*	2	0.159
P10	BALF	*Klebsiella aerogenes*	Negative	*Stenotrophomonas maltophilia*	364	1.221
		*Candida albicans*		*Klebsiella aerogenes*	156	0.444
		(BALF)		*Candida albicans*	3380	Copies/ml
				*Cytomegalovirus*	61	3.761
P11	BALF	Negative (BALF and blood)	Negative	*Haemophilus parainfluenzae*	6	0.100
				*Sphingomonas paucimobilis*	2	0.007
				*Escherichia coli*	2	0.005
P12	Pus	*Staphylococcus aureus* (Pus)	Negative	*Stenomonas maltophilia*	19	0.03
				*Staphylococcus aureus*	5	0.02
P13	sputum	Multiple drug resistant pseudomonas aeruginosa (Sputum)	Negative	*Pseudomonas aeruginosa*	1599	3.572
				*Torque teno virus*	56	13.687
				*Epstein-barr virus*	376	10.228
				*Cytomegalovirus*	23	1.378
P14	Bone biopsy tissue	Negative	PPD (+)	*Nocardia farcinica*	752	0.73
		(Blood)	T-SPOT (+)	*Mycobacterium avium*	2	0.01
P15	CSF	Negative (CSF and blood)	Negative	Negative	/	/
P16	CSF	Negative	Sputum X-pert (+)	*Mycobacterium*	2	0.01
		(CSF and blood)		*Rasamsonia emersonii*	1	0.01

CMV, Cytomegalovirus; EBV, Epstein-barr virus; SMRN, strictly map reads number.

**Table 2:** Comparison of positive results and agreement among mNGS, CMT and culture method in patients.

Group	mNGS-positive	mNGS-negative	Total number
CMT-positive	11	0	11
CMT-negative	4	1	5
Culture-positive	5	0	5
Culture-negative	10	1	11
Total number	15	1	16

### Distribution of Identified Pathogens

**Figure 3:**
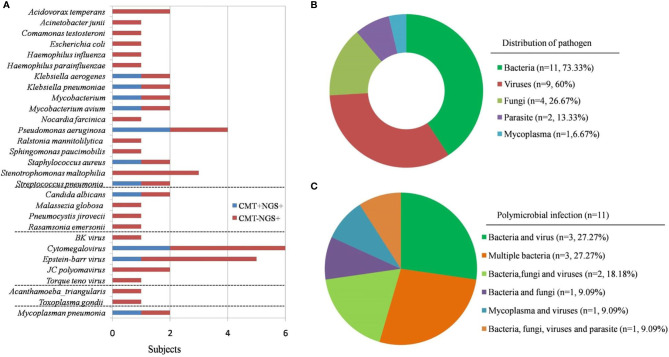
Pathogens identified in PID patients using CMT and NGS. **(A)** The figure shows the number of subjects in whom each microbe was detected. Blue bars indicate microbes detected by CMT and predicted to be pathogens by NGS (CMT+NGS+). Red bars indicate microbes detected by NGS only (CMT−NGS+). **(B)** Distribution of pathogens in patients with PID. **(C)** Polymicrobial infection accounted for 73.33% of cases; different kinds of polymicrobial infection are shown.

In the present study, the diagnosis of 15 patients was confirmed by both clinical and microbiological criteria. According to the results, bacteria (73.33%) were the most common pathogens identified, followed by virus (60%), fungi (26.67%), and parasites (13.33%). It is noteworthy that among the 15 patients with identified pathogens, 11 were diagnosed with polymicrobial infections (73.33%). In particular, Cytomegalovirus (CMV) and Epstein-Barr virus (4/15) were the most common pathogens, followed by *Stenomonas maltophilia* (3/15) (**Figure 3**).

The following pathogens were identified in different types of PID patients. Among the four patients with XHIM, two patients had *JC polyomavirus* infection, one patient had toxoplasmosis infection, and one had a mixed infection by *Stenotrophomonas maltophilia, Pneumocystis jirovecii*, and CMV. Both patients with WAS were infected with human herpesviruses, one with EBV and another with CMV. Mixed bacterial infections (including opportunistic pathogens such as *Sphingomonas paucimobilis* and *Stenomonas maltophilia*) were identified in both patients with CGD. Others included *Mycobacterium* (in a patient with APDS), and *Nocardia farcinica* and *Mycobacterium avium* mixed infection (in a patient with MSMD) (**Table 1**).

### Comparison of mNGS With CMT

**Figure 4:**
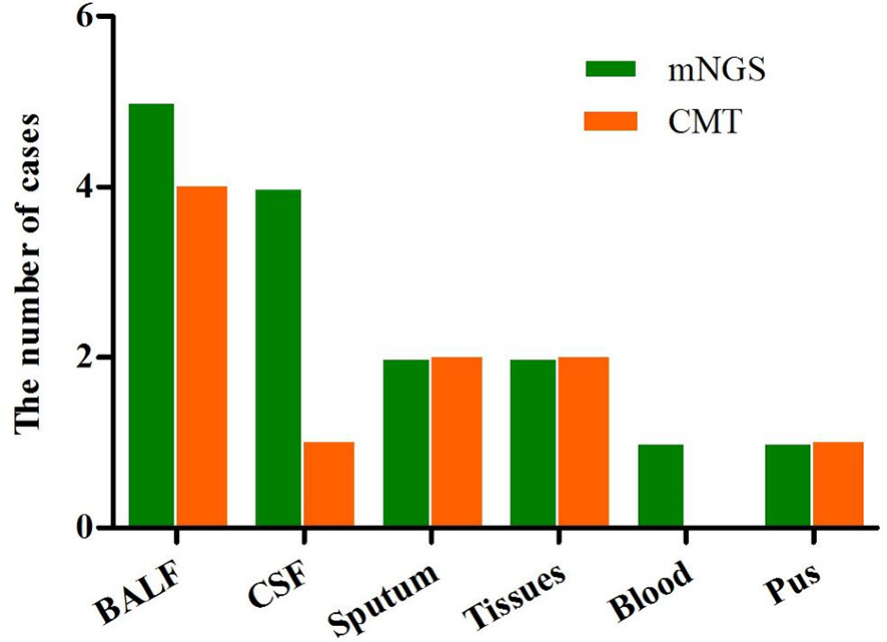
Overall sensitivity of mNGS and CMT in the different sample types.

In CSF, BALF, and blood samples, mNGS showed higher sensitivity for microbes detection than CMT; however, there was no difference with respect to sputum, tissue, and pus (**Figure 4**). In samples from 11 patients with polymicrobial infections, multiple microbiological tests were performed to obtain a final pathogenic diagnosis; these were more time-consuming and costly than tests used for monomicrobial infections. However, mNGS yielded consistent results from only one test in 11 of the 15 subjects. We analyzed the time taken to determine the pathogenic diagnosis. For monomicrobial infections, the time for mNGS and CMT was not statistically different; however, for polymicrobial infections, mNGS required significantly less time to identify the pathogens than CMT (*P* < 0.05). In terms of testing time, CMT for the 11 patients took 3–7 days, while the sequencing time was only 48 hours. In terms of detection efficiency, five of the 16 patients were negative by CMT, whereas mNGS detected pathogens in 15 patients; some of these had a mixed infection, suggesting that mNGS has better detection efficiency.

### Treatment and Follow-Up

Of the 16 patients, P3 improved significantly after treatment with sulfamethoxazole. P4 improved after treatment with sulfamethoxazole and ganciclovir; P5 and P7–P11 were treated successfully with anti-infection therapy, including antibiotics and antifungal agents. P12 was treated successfully with sulfamethoxazole, imipenem cilastatin, and linezolid. P14 improved after treatment with isoniazid, rifampicin, pyrazinamide, ethambutol, and sulfamethoxazole. P16 improved after treatment with isoniazid, rifampicin, pyrazinamide, ethambutol, and linezolid. P1 and P2 died within 1 year of *JC virus* infection. Pathological analysis of the liver lesions in patient P6 were diagnosed as EBV-associated leiomyosarcoma; the patient is still alive and awaiting HSCT. P13 died from severe infection caused by multi-drug resistant *Pseudomonas aeruginosa.* Some patients (P1-P6, P10-P13, P16) routinely received oral sulfamethoxazole to prevent *pneumocystis carinii pneumonia* or bacterial infection. In P11 and P12, itraconazole was taken orally to prevent fungal infection.

## Discussion

There are several causes why it is difficult to identify pathogens in PID. First, some patients have received antibiotics empirically before pathogen identification, thereby reducing the chances of a positive culture. Second, multiple concurrent infections and atypical pathogens are common in patients with PID; therefore, it can be difficult to identify the target pathogen using traditional cultures, PCR, immunofluorescence analysis, serological tests, and other CMT. Furthermore, because a considerable number of patients with PID are deficient in antibody production, antibody-dependent detection methods are ineffective. Also, infection-related clinical and imaging manifestations may appear atypical in patients with PID. These characteristics pose a severe challenge to pediatricians, or even PID experts, with respect to establishing specific etiology and choosing appropriate tests. Therefore, rapid and effective laboratory tests are necessary.

Reports on the use of mNGS to identify infections in patients with PID are scarce, although many previous studies recommend the use of mNGS for pathogen detection in patients with secondary immunodeficiency disease and in intensive care patients with life-threatening infections (5, 12–14). In November 2020, Chinese experts published an expert consensus on the clinical application of China’s mNGS technology for detecting infectious pathogens. They pointed out that for new, rare, and treatment-refractory infectious diseases, and for patients with immunocompromising disease, mNGS can improve the pathogen detection rate significantly and can be used as the first-line method of detection (19).

Several studies report low positivity rates by conventional methods such as culture (20, 21). This study highlight the capacity of mNGS to detect pathogens that are unidentifiable by CMT. For example, P1–P3 had a suspected CNS infection, although no pathogens were detected by CMT. However, mNGS identified *JC virus* and *toxoplasmosis*. Moreover, most patients with no pathogenic evidence of infection had received empirical antibiotics. This led to side effects such as intestinal dysbacteriosis, liver and kidney damage. The high incidence of *Stenotrophomonas maltophilia* (3/15) in this study maybe evidence for carbapenem abuse, in addition to immunodeficiency itself. Therefore, mNGS may be used to exclude fever as a sign of infection. Implementation of mNGS as a rule-out strategy may reduce the abuse of antibiotics, as well as the duration of antibiotic therapy, in these patients.

Previous studies demonstrate the utility of mNGS for detecting pathogens that cause (or may cause) encephalitis, bloodstream infections, lower respiratory tract infections, and focal infections, in different sample types (5–14). Multiple case reports describe the use of mNGS to identify viruses, bacteria, fungi, and parasites in CSF and brain tissue (8, 10). A previous study shows that compared with CMT, mNGS has a sensitivity of 73%, a specificity of 99%, a positive predictive value of 81%, and a negative predictive value of 99%, for pathogen detection in CSF (22). Another multicenter study found that mNGS of CSF samples represents a potential step forward in the diagnosis of meningoencephalitis, thereby guiding earlier and more targeted treatments for neuroinvasive infections, and identifying emerging infections and disease phenotypes (9). In terms of the effectiveness and sensitivity of mNGS in different samples, we found that the sensitivity of mNGS for detecting pathogens in CSF, BALF, and blood samples was higher than that of CMT. Also, a recent study showed that mNGS is more sensitive than CMT for detecting pathogens in BALF, tissue, blood, and sputum samples (23).

Several studies report that polymicrobial infections are one of the most important features of immunocompromised hosts (24–26). Several studies highlight the potential of mNGS to supplement routine diagnostic methods in cases of co-infection with multiple pathogens (5, 12, 13). Consistent with previously studies, we found that 11 patients had polymicrobial infections; in our hands, mNGS showed clear advantages over other methods with respect to high detection efficiency and speed (results obtained in 48 h).

Patients with PID are susceptible to infection by opportunistic pathogens or rare pathogens (1, 2). We found that mNGS was superior to CMT for identification of opportunistic pathogenic microorganisms such as *JC polyomavirus*, *Pneumocystis jirovecii*, and *Stenotrophomonas maltophilia*, and for detection of causative agents that either had a relatively low culture rate or took a long time to culture (e.g., *Mycobacterium*). *Stenotrophomonas maltophilia* is a common nosocomial opportunistic pathogen in immunocompromised patients and in patients with Job’s syndrome (27, 28). In the present study, P12 developed a liver abscess caused by co-infection by *Stenotrophomonas maltophilia* and *Staphylococcus aureus*; and was relieved by treatment with linezolid and compound Sulfamethoxazole. Toxoplasmic encephalitis (TE) is one of the most important neurological opportunistic infections in T cell-deficient patients. *Toxoplasma gondii* is difficult to culture and microscopic examination of CSF is insensitive. TE can be identified by cranial imaging, PCR, and antibody detection methods, whereas it highly depends on the doctor’s experience. In addition, the serum anti-toxoplasma antibody is always absent in PID patients (8). In patient P3, we diagnosed TE by mNGS; this patient recovered after targeted sulfamethoxazole treatment. The number of standard unique reads for *Mycobacterium* and *Pneumocystis jirovecii* was lower than that for other pathogens. It is difficult to obtain circulating genomic DNA from intracellular bacteria (29); thus, low reads can also provide clues for diagnosis. A previous study demonstrates that for patients suspected of having TB, the diagnostic ability of mNGS was similar to that of Xpert; and mNGS may be more suitable for scarce samples such as CSF (30).

However, mNGS still has some limitations. The host genetic background and the background caused by bacterial contamination creates “noise”, there are no uniform standards for detailed experimental procedures, it is difficult to distinguish infection from colonization, there is a lack of standardization with bioinformatics analysis processes regarding cut-off values, and data interpretation is tricky (22). It is extremely challenging to confirm the true pathogenicity of multiple pathogen infections, or opportunistic pathogen infections, detected by mNGS in patients with PID. In fact, the CMT also facing this problem. The microbe results must be interpreted in the context of the clinical situation, including immune defect itself, clinical manifestations and therapeutic effects.

This study has certain limitations. First, the sample size was relatively small, and we did not categorize specific types of PID as such a rare disease. Further studies should recruit a larger cohort and categorize patients with specific types of PID to better explore and describe the pathogen spectrum and its relationship to deficiency subtype. Second, some of the patients in our study received antibiotics therapy, which will affect the diagnostic performance of both mNGS and CMT. Additionally, RNA was not sequenced in all specimens for economic factors and CMT include common RNA virus testing; therefore, rare causes of infection (such as RNA viruses) may have been partially missed. Finally, this was a single center retrospective study so there is potential for bias. Besides, it is often unclear whether microbes detected using mGNS are contaminant, colonizer or pathogen, which need more study especially in PID.

In conclusion, our data suggest that mNGS results showed higher agreement with the clinical diagnosis and took shorter time. mNGS is a strong candidate for pathogenic diagnosis in PID patients with suspected severe, mixed, complicated, or treatment-refractory infections. The mNGS technology has marked diagnostic potential for complementing routine diagnostic methods, particularly in the context of opportunistic pathogens and mixed infections, or in cases with negative CMT results. However, the identification of pollutants, colonizers or pathogens still needs more study, which is also a problem faced by CMT.

## Data Availability Statement

The raw sequence data reported in this paper have been deposited in the Genome Sequence Archive (Genomics, Proteomics & Bioinformatics 2017) in National Genomics Data Center (Nucleic Acids Res 2021), China National Center for Bioinformation / Beijing Institute of Genomics, Chinese Academy of Sciences, under accession number CRA004615 that are publicly accessible at https://ngdc.cncb.ac.cn/gsa.

## Ethics Statement

The studies involving human participants were reviewed and approved by Medical Ethics Committee of the Children’s Hospital of Chongqing Medical University. Written informed consent to participate in this study was provided by the participants’ legal guardian/next of kin.

## Author Contributions

WT planned the study, analyzed the data, and wrote the paper. YA conceived and designed the study, supervised the study, and revised the paper. YZ, CL, LZ, and ZZ collected samples and carried out clinical diagnosis, treatment and follow up of patients. XT and XZ revised the paper. All authors contributed to the article and approved the submitted version.

## Conflict of Interest

The authors declare that the research was conducted in the absence of any commercial or financial relationships that could be construed as a potential conflict of interest.

## Publisher’s Note

All claims expressed in this article are solely those of the authors and do not necessarily represent those of their affiliated organizations, or those of the publisher, the editors and the reviewers. Any product that may be evaluated in this article, or claim that may be made by its manufacturer, is not guaranteed or endorsed by the publisher.
