# Supplementary material for: Clinical Application of Metagenomic Next-Generation Sequencing for Suspected Infections in Patients With Primary Immunodeficiency Disease
Source: Front Immunol. 2021 Aug 13;12:696403. doi: 10.3389/fimmu.2021.696403 (PMC8414648; doi:10.3389/fimmu.2021.696403)
Supplement: Supplementary file 1 [file Table_1.doc]

**TABLE 1** Clinical and laboratory characteristics of patients included

ALC, absolute lymphocyte count; IVIG, intravenous immunoglobulin; CNS, central nervous system; XHIM, X-linked-hyper IgM syndrome; WAS, Wiskott-Aldrich syndrome; PAMI, PSTPIP1-associated myeloid-related proteinemia inflammatory syndrome; XLP, X-linked lymphoproliferative disease; CGD, chronic granulomatous disease; MSMD, Mendelian susceptibility to mycobacterial disease; XLA, X-linked agammaglobulinemia; APDS, activated phosphoinositide 3-kinase-δ syndrome.

| Patients | Age  （years） | Gender | WBC  （*10^9^/L） | ALC  （*10^9^/L） | Lymphocyte Subsets | Immunoglobulins | CRP（mg/L）/  PCT（ng/ml） | Suspected infectious  Disease | PID | Gene mutation |
| --- | --- | --- | --- | --- | --- | --- | --- | --- | --- | --- |
| P1 | 12.25 | Male | 4.36 | 1 | CD3, 34.79%; CD19, 33.8%;  CD4, 19.01%; CD8, 9.86%;  CD56+16, 30.7% | IgG 4.16 g/L; IgM 3.96 g/L  IgA 0.88 g/L; IgE 3 IU/ml  (after IVIG) | <8  0.178 | CNS infection | XHIM | CD40LG  c.654C>A  p.C218X |
| P2 | 12 | Male | 4.84 | 3.87 | CD3, 75.5%; CD19, 17.0%;  CD4, 34.1%; CD8, 33.2%;  CD56+16, 6.4%. | IgG 8.96 g/L; IgM 2.39 g/L  IgA<0.067g/L; IgE0.3 IU/ml  (after IVIG) | 9  0.016 | CNS infection | XHIM | CD40LG  c.158-161delTAGA  p.I53 fs X13 |
| P3 | 16 | Male | 6.06 | 2.48 | CD3, 76.3%; CD19,15.7%;  CD4, 33.8%; CD8, 37.6%;  CD56+16, 7.2%. | IgG 12.7 g/L; IgM 1.41 g/L  IgA<0.067 g/L; IgE<5 IU/ml  (after IVIG) | <8  0.036 | CNS infection | XHIM | CD40LG  exon 4 and exon 5 deletion |
| P4 | 0.33 | Male | 14.78 | 8.28 | CD3, 80.4%; CD19,14.66%;  CD4, 48.5%; CD8, 31.3%;  CD56+16, 4.9%. | IgG 0.78 g/L; IgM 0.517 g/L  IgA<0.067g/L; IgE<5 IU/ml | <8  0.055 | pneumonia | XHIM | CD40LG  c.654C>A  p.C218X |
| P5 | 0.67 | Male | 9.04 | 5.06 | CD3, 58.2%; CD19,14.5%;  CD4, 23.9%; CD8, 32.5%;  CD56+16, 26.9%. | IgG 17.7g/L; IgM 2.54 g/L  IgA 0.46g/L; IgE 3210 IU/ml  (after IVIG) | 50  0.082 | sepsis | WAS | WAS  c.777+3_777+6 del GAGT  splicing |
| P6 | 6.67 | Male | 21.37 | 1.45 | CD3, 55.24%;CD19,12.59%;  CD4, 27.35%; CD8, 19.48%;  CD56+16, 29.44%. | IgG 5.03g/L; IgM 0.217 g/L  IgA 0.98g/L; IgE 49.7 IU/ml  (after IVIG) | 81  7.11 | Liver special pathogen infection | WAS | WAS  c.290G>A  p.W97X |
| P7 | 8.92 | Male | 11.78 | 4.01 | CD3, 66.92%; CD19, 9.14%;  CD4, 38.97%; CD8, 22.17%;  CD56+16, 23.37%. | IgG 5.81g/L; IgM 1.09 g/L  IgA 1.43g/L; IgE 38.5IU/ml | <8  / | pneumonia | CTLA4 deficiency | CTLA4  c.208C>T  p.R70W |
| P8 | 2.33 | Male | 2.59 | 1.68 | CD3, 84.42%; CD19, 7.10%;  CD4, 32.73%; CD8, 49.22%;  CD56+16, 4.47%. | IgG 11.4g/L; IgM 1.22 g/L  IgA 2.35g/L; IgE 313IU/ml | 106  1.17 | pneumonia | PAMI | PSTPIP1  c.708 C>G  p.N236K |
| P9 | 9.75 | Male | 14.46 | 3.9 | CD3, 90.04%; CD19,1.93%;  CD4, 43.47%; CD8, 42.07%;  CD56+16, 8.01%. | IgG 11.4g/L; IgM 0.062 g/L  IgA<0.067g/L; IgE 0.8IU/ml  (after IVIG) | 26  0.126 | pneumonia | XLP | SH2D1A  c.163C>T  p.R55X |
| P10 | 0.33 | Female | 10.9 | 6.65 | CD3, 92.76%; CD19,5.03%;  CD4, 21.87%; CD8, 70.14%;  CD56+16, 2.20%. | IgG 3.56 g/L; IgM 0.67 g/L  IgA<0.067g/L; IgE 5 IU/ml | 11  0.56 | pneumonia | NEMO  deficiency | NFKBIA  c.40 G>T  p.E14X |
| P11 | 2.58 | Male | 22.46 | 4.49 | CD3,60.17%; CD19,29.66%;  CD4, 27.61%; CD8, 29.43%;  CD56+16, 9.6%. | IgG 18.6g/L; IgM 1.23 g/L  IgA 2.46g/L; IgE 415IU/ml | 102  0.173 | pneumonia | CGD | CYBB  c.613delT  p.R226 fs X5 |
| P12 | 14.33 | Male | 12.93 | 1.81 | CD3,79.38%; CD19,15.32%;  CD4,28.61%; CD8, 45.74%;  CD56+16, 5.21%. | IgG 19.1g/L; IgM 0.755g/L  IgA3.72g/L; IgE 81.8IU/ml | 87  0.805 | Liver abscess | CGD | CYBB  c.804+1G>A  p.W206 fs X8 |
| P13 | 6.17 | Male | 8.24 | 1.32 | CD3,63.68%; CD19,0.71%;  CD4,26.94%; CD8, 25.53%;  CD56+16, 34.52%. | IgG 3.85g/L; IgM 0.951g/L  IgA<0.067g/L; IgE 0.8IU/ml  (after IVIG) | 37  0.056 | pneumonia | Artemis deficiency | DCLRE1C  c.679-1G＞T, splicing  c.419C＞T, p.A140V |
| P14 | 2.58 | Male | 10.47 | 3.35 | CD3,64.11%; CD19,18.05%;  CD4,29.91%; CD8,26.26%;  CD56+16,17.30%. | IgG18.2g/L; IgM 2.37g/L  IgA3.34g/L; IgE 5.9IU/ml | 29  0.65 | bone special pathogen infection | MSMD | STAT1  c.736G>A  p.A246T |
| P15 | 3.83 | Male | 12.75 | 6.12 | CD3,95.79%; CD19,0.04%;  CD4,58.03%; CD8,30.56%;  CD56+16,3.93%. | IgG 9.59g/L; IgM<0.042g/L  IgA<0.067g/L; IgE <5IU/ml  (after IVIG) | <8  0.23 | CNS infection | XLA | BTK  c.37C>T  p.R13X |
| P16 | 14 | Male | 5.23 | 0.47 | CD3, 89.74%; CD19,8.17%;  CD4,19.82%; CD8,63.85%;  CD56+16,2.0%. | IgG 16.4g/L; IgM 6.85g/L  IgA 1.01g/L; IgE <5 IU/ml | 9  0.08 | CNS infection | APDS | PIK3CD  c.3061G>A  p.E1021K |
